# Comparison of PETINIA and LC-MS/MS for determining plasma mycophenolic acid concentrations in Japanese lung transplant recipients

**DOI:** 10.1186/s40780-018-0101-7

**Published:** 2018-04-02

**Authors:** Masafumi Kikuchi, Masaki Tanaka, Shinya Takasaki, Akiko Takahashi, Miki Akiba, Yasushi Matsuda, Masafumi Noda, Kanehiko Hisamichi, Hiroaki Yamaguchi, Yoshinori Okada, Nariyasu Mano

**Affiliations:** 1 Department of Pharmaceutical Sciences, Tohoku University Hospital, 1-1 Seiryo-machi, Aoba-ku, Sendai, 980-8574 Japan; 2 Department of Organ Transplantation Center, Tohoku University Hospital, 1-1 Seiryo-machi, Aoba-ku, Sendai, 980-8574 Japan; 3 Department of Thoracic Surgery, Tohoku University Hospital, 1-1 Seiryo-machi, Aoba-ku, Sendai, 980-8574 Japan

**Keywords:** Mycophenolic acid, Mycophenolic acid acyl glucuronide, Japanese, Lung transplant, PETINIA, LC-MS/MS, Positive bias

## Abstract

**Background:**

Mycophenolic acid (MPA) treatment requires therapeutic drug monitoring to improve the outcome after organ transplantation. The aim of this study was to compare two methods, a particle enhanced turbidimetric inhibition immunoassay (PETINIA) and a reference liquid chromatography tandem mass spectrometry (LC-MS/MS) for determining plasma MPA concentrations from Japanese lung transplant recipients.

**Methods:**

Plasma MPA concentrations were determined from 20 Japanese lung transplant recipients using LC-MS/MS and the PETINIA on the Dimension Xpand Plus-HM analyzer.

**Results:**

The mean MPA concentration measured by PETINIA was significantly higher than that measured by LC-MS/MS (3.26 ± 2.73 μg/mL versus 2.82 ± 2.71 μg/mL, *P* < 0.0001). The result of the Passing Bablok analysis was a slope of 1.104 (95% confidence interval [CI], 1.036–1.150) and an intercept of 0.229 (95%CI, 0.144–0.315). Bland–Altman analysis revealed PETINIA overestimates plasma MPA concentration by 26.25% and 95%CI from 21.43 to 31.07%.

**Conclusion:**

The measurement of MPA by the PETINIA in Japanese lung transplant patients should evaluate the result with attention to positive bias.

## Background

Mycophenolate mofetil (MMF) is an orally ingestible prodrug of mycophenolic acid (MPA), which exerts immunosuppressive activity. MMF is widely used as an immunosuppressant to prevent rejection after organ transplantation [1]. Dose adjustment of MMF based on plasma MPA concentration has been reported to improve therapeutic performance [2]. Therefore, accurate analytical methods are necessary to measure plasma MPA concentration. MPA in plasma can be determined by high performance liquid chromatography combined with ultraviolet detection (HPLC-UV), liquid chromatography-tandem mass spectrometry (LC-MS/MS), or automated immunoassays [3, 4]. Although immunoassays are widely used in clinical laboratories due to ease of adopting such methods on automated analyzers, both immunoassays and chromatographic methods are available for therapeutic drug monitoring of MPA. Recently, Siemens Healthcare Diagnostics (Newark, DE, USA) developed a particle-enhanced turbidimetric inhibition immunoassay (PETINIA) to determine MPA (Flex reagent cartridge) using the Dimension analyzers. As PETINIA shows cross-reactivity with the pharmacologically active mycophenolic acid acyl glucuronide (AcMPAG), plasma MPA concentration is often overestimated [package insert] [5]. In fact, plasma MPA concentration in the liver, kidney, and heart transplant patients has been reported to exhibit a positive bias [6, 7]. On the other hand, although the enzyme multiplied immunoassay technique (EMIT) also shows cross-reactivity with AcMPAG, it has been reported that MPA concentration showed almost no positive bias in islet transplant patients, or positive bias in liver transplantation or kidney transplantation [7, 8]. In addition, the pharmacokinetics of MPA and its metabolites are reported to be different between lung transplant patients and heart transplant patients [9]. Moreover, it has been reported that MPAG clearance in Caucasians is significantly higher than that in Chinese subjects, suggesting pharmacokinetic differences between races [10]. Therefore, the pharmacokinetics of MPA and its metabolites is presumed to differ for each transplanted organ and race. However, limited data exist for plasma MPA concentration by PETINIA, and have not been reported for Japanese patients or lung transplantation recipients. The aim of this study was to compare MPA concentrations obtained by PETINIA and LC-MS/MS, as a reference method, using samples from Japanese lung transplant recipients.

## Methods

### Study design

This study was prospectively designed, and all procedures were approved by the Ethics Committee of Tohoku University Graduate School of Medicine (2015–1-150). Plasma samples were collected from patients at Tohoku University Hospital after receiving informed consent.

### Immunosuppressive regimen

MMF was orally administered twice daily on empty stomach. In patients who received 250 mg/day of MMF, it was administered only once daily to avoid adverse effects. MMF was used in combination with tacrolimus (TAC) or cyclosporine (CyA) and prednisolone to maintain immunosuppression.

### Clinical sample collection

Blood samples were obtained, before, 0.5 h after, and 2 h after MMF administration, from Japanese lung transplant patients. Blood samples were collected in 3 mL heparinized collection tubes, and centrifuged at 1580 *g* for 10 min at 4 °C to obtain the plasma. All plasma samples were stored at − 40 °C until measurement. MPA concentration was determined by using PETINIA and LC-MS/MS on the same day.

### LC-MS/MS (reference method)

We used a slightly modified-method based on the report by Md Dom et al. and Kawanishi et al. [11, 12]. Thirty microliters of plasma was mixed with 30 μL of 1 μg/mL mycophenolic acid-d3 (MPA-D_3_), the internal standard, and 120 μL of acetonitrile. The mixture was vortex-mixed for 1 min and centrifuged at 15,100 *g* for 5 min. Next, 100 μL of the supernatant was diluted 1:2 with water and a 3 μL aliquot was injected into the LC-MS/MS system (LCMS-8050; Shimadzu, Kyoto, Japan). The LC-MS/MS system was operated in multiple reaction monitoring mode with positive-ion detection mode. The temperatures of the columns and autosampler were maintained at 40 °C and 4 °C, respectively. LabSolutions LCMS software (Shimadzu) was used to control the instruments and to process the data. For MPA analysis, chromatographic separation was achieved using a Shim-pack GIS (75 mm × 2.1 mm i.d., particle size 3 μm: Shimadzu GLC, Tokyo, Japan) under isocratic elution conditions, with a mobile phase consisting of 1% acetic acid in water/acetonitrile (1:1, v/v) at a flow rate of 0.3 mL/min. The retention times were as follows: MPA, 1.75 min; and MPA-D_3_, 1.75 min. Molecules were detected according to the following mass transitions: m/z 321.4 > 207.3 (MPA); and m/z 324.4 > 210.3 (MPA-D_3_). We used six calibrators with concentrations of 0.06, 0.2, 0.6, 2, 6, and 20.0 μg/mL for developing a calibration curve. The lower limit of quantitation was defined as the lowest concentration with a signal-to-noise ratio of at least 10. Three controls (0.1, 5, and 15 μg/mL) were used for quality control. Linearity was achieved with a correlation coefficient of 0.999. The intra-day and inter-day precisions were less than 3.7%, and accuracy was within ±8.5%.

### PETINIA

The mycophenolic acid assay kits (Dimension® MPAT Flex® reagent cartridge), calibrators, and controls were provided by Siemens Healthcare Diagnostics (Newark, DE, USA), and assays were run using a Dimension Xpand Plus-HM analyzer, also obtained from Siemens Healthcare Diagnostics. The assay was based on a direct, fully automated method, as per the manufacturer’s instructions, and was valid from 0.2 to 30.0 μg/mL.

### Statistical analysis

The comparison between PETINIA and LC-MS/MS was performed using the Wilcoxon signed rank test. Consistency of the results was analyzed using Passing–Bablok regression analysis and Bland-Altman difference plots. *P*-values of < 0.05 were considered to indicate a significant difference for the above tests.

## Results

**Fig. 1:**
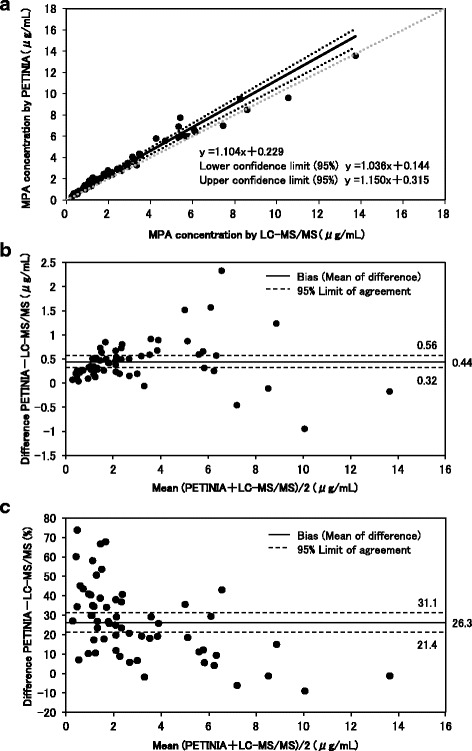
Comparison of MPA concentration from PETINIA and LC-MS/MS assays using Passing-Bablok regression analysis (**a**) and Bland-Altman difference plots (**b**, **c**)

A total of 60 plasma samples from 20 Japanese lung transplant patients (5 men and 15 women; median age 49 (23–65) years; median of 2.7 (0.3–15.1) years post-lung transplant) were available for this study. The median (range) MMF dose was 750 (250–1500) mg/day, and MMF was combined with TAC and CyA in 85% and 15% of the patients, respectively. The median (range) serum albumin concentration was 4.0 (2.6–4.4) g/dL, aspartate aminotransferase 15 (9–23) IU/L, alanine aminotransferase 10 (5–48) IU/L, and estimated glomerular filtration rate (eGFR) 53 (34–112) mL/min/1.73m^2^. The mean MPA concentration measured by PETINIA was significantly higher than that measured by LC-MS/MS (3.26 ± 2.73 μg/mL versus 2.82 ± 2.71 μg/mL, *P* < 0.0001) (Table 1). Although regression analysis showed a strong linear relationship (r^2^ = 0.969, *P* < 0.0001) between PETINIA and LC-MS/MS, the result of the Passing–Bablok analysis indicated a systematic difference with a slope of 1.104 (95% confidence interval [CI], 1.036–1.150) and an intercept of 0.229 (95%CI, 0.144–0.315) (Fig.1a). The Bland–Altman analysis revealed a mean bias of 0.44 μg/mL (95%CI, 0.32–0.56), comprising 26.25% (95%CI, 21.43–31.07) (Fig. 1b, c). Patients were divided among 7 groups with normal or mild loss of kidney function (median (range) eGFR of 76 (69–112) mL/min/1.73m^2^), and 13 groups with mild-to-moderate or moderate-to-severe loss of kidney function (median (range) eGFR of 48 (34–57) mL/min/1.73m^2^). The Bland–Altman analysis of patients with normal or mild loss of kidney function revealed a mean bias of 28.13% (95%CI, 19.61–36.65). The Bland–Altman analysis in patients with mild-to-moderate or moderate-to-severe loss of kidney function revealed a mean bias of 25.14% (95%CI, 19.06–31.22) (data not shown). Plasma MPA concentration increased in accordance with sampling time (C0, C0.5, C2), but no change in absolute bias was observed. Thirty-six measures (60%) showed positive bias over 20% (data not shown).

**Table 1:** MPA concentration measured by PETINIA and LC-MS/MS by sampling times

Sampling Time (h)	Number	MPA Concentration Measured by PETINIA, Mean ± SD (μg/mL)	MPA Concentration Measured by LC-MS/MS, Mean ± SD (μg/mL)	Wilcoxon signed rank test
0	20	1.89±1.35	1.46±1.05	*P* < 0.0001
0.5	20	3.24±3.02	2.82±3.07	*P* < 0.0001
2	20	4.64±2.87	4.17±2.91	*P* = 0.0024
0, 0.5, 2	60	3.26±2.73	2.82±2.71	*P* < 0.0001
Sampling Time (h)	Passing–Bablok		Bland–Altman	
	Slope (95% CI)	Intercept (95% CI)	Bias (95% CI) (μg/mL)	Bias (95% CI) (%)
0	1.242 (1.131–1.392)	0.054 (− 0.139–0.193)	0.44 (0.27–0.60)	31.83 (24.37–39.28)
0.5	1.014 (0.944–1.153)	0.306 (0.141–0.483)	0.42 (0.25–0.59)	28.19 (18.31–38.06)
2	1.044 (0.920–1.126)	0.417 (0.185–0.692)	0.47 (0.17–0.78)	18.54 (10.69–26.40)
0, 0.5, 2	1.104 (1.036–1.150)	0.229 (0.144–0.315)	0.44 (0.32–0.56)	26.25 (21.43–31.07)

## Discussion

This is the first study to compare PETINIA and LC-MS/MS in determining plasma MPA concentrations in Japanese patients and lung transplant recipients. A PETINIA method overestimates plasma MPA concentration due to cross-reactivity of AcMPAG, and is known to have an average value of approximately 50% (package insert). Hence, the AcMPAG cross-reactivity greatly influenced plasma MPA concentration, even in Japanese lung transplant patients. Our study revealed an average positive bias of 0.44 μg/mL (26.3%) in the PETINIA analysis compared to that with LC-MS/MS in lung transplant patients. Dasgupta et al. observed an average positive bias of 22.4% in 18 liver transplant patients and 8.3% in 42 kidney transplants in MPA concentrations determined by PETINIA compared with values obtained by HPLC-UV [6]. In a similar setting, Kunicki et al. observed an average bias of 33.5% in 192 heart transplant patients [7]. The result of this study was the second highest average positive bias after that found in heart transplantation patients. Although research on liver, kidney, and heart transplant patients does not describe kidney function, there were many patients with decreased kidney function due to adverse effects of TAC or CyA in this study. Previously, González-Roncero et al. reported that MMF dose or MPA 12-hour area under the concentration-time curve (AUC_0–12_) values were not affected by kidney function, but mean pre-dose levels of AcMPAG-C0 and AcMPAG-AUC_0–12_ were much higher in patients with advanced kidney insufficiency than in those with preserved kidney function [13]. However, there was no difference in average positive bias between patients with normal or mild loss of kidney function, and mild-to-moderate or moderate-to-severe loss of kidney function in this study. Therefore, this large positive bias in Japanese lung transplant patients may be related to factors such as transplanted organ and race.

Although there was almost no difference in absolute bias between sampling times (C0, C0.5, C2), the absolute percentage bias was found to be different from 31.83%, 28.19%, and 18.54%, respectively. In vitro research suggests that TAC increases blood MPA levels by inhibiting the UDP-glucuronosyltransferase involved in MPA metabolism [14, 15]. Alternatively, Deters et al. postulated that CyA significantly reduces serum MPA levels by suppressing biliary excretion of 7-O-mycophenolic acid glucuronide (MPAG) and decreasing MPA recirculation [16]. Moreover, Shipkova et al. suggested that administration of CyA increases the blood concentration of MPAG and AcMPAG [17]. In this study, the number of combined uses of MPA and TAC was very high in 17 of 20 cases. Therefore, it was considered that the difference in absolute bias between the sampling times was small.

As EMIT also shows cross-reactivity with AcMPAG, plasma MPA concentration is known to be overestimated [7]. Therefore, the guidelines on therapeutic drug monitoring of immunosuppressive drugs in organ transplantation recommend that AUC_0–12_ values in the early posttransplant stages be maintained within the therapeutic window of 30–60 μg**·**h/mL for HPLC, or 35–70 μg**·**h/mL for EMIT. It is recommended the target pre-dose concentration be maintained within the range of 1–3.5 μg/mL for HPLC and 1.3–4.5 μg/mL for EMIT [18]. Although the target AUC_0–12_ value and pre-dose concentration by PETINIA is not described in the guideline, it should be maintained higher than HPLC in Japanese lung transplant patients.

## Conclusion

Measurement results of MPA by the PETINIA method in Japanese lung transplant patients should be evaluated with attention to positive bias.

## Abbreviations

AcMPAG: Mycophenolic acid acyl glucuronide
AUC: Area under the concentration-time curve
CI: Confidence interval
CyA: Cyclosporine
eGFR: Estimated glomerular filtration rate
EMIT: Enzyme multiplied immunoassay technique
HPLC-UV: High performance liquid chromatography combined with ultraviolet detection
LC-MS/MS: Liquid chromatography tandem mass spectrometry
MMF: Mycophenolate mofetil
MPA: Mycophenolic acid
MPAG: 7-O-mycophenolic acid glucuronide
PETINIA: Particle Enhanced Turbidimetric Inhibition Immunoassay
TAC: Tacrolimus
